# Comparison of Clinical and Functional Outcomes in Anterior Cruciate Ligament Reconstruction Using Peroneus Longus Tendon Versus Hamstring Tendon Autograft: A Prospective Comparative Study

**DOI:** 10.7759/cureus.102438

**Published:** 2026-01-27

**Authors:** Saurav Soni, Jagdeep Rehncy, Dharminder Singh, Sandeep Kumar, Harsh Kumar, Girish Sahni, Gurpreet Singh

**Affiliations:** 1 Orthopaedics, Government Medical College and Rajindra Hospital, Patiala, Patiala, IND; 2 Orthopaedics, Government Medical College, Patiala, Patiala, IND

**Keywords:** anterior cruciate ligament reconstruction, donor site morbidity, functional outcomes, graft diameter, hamstring tendon, knee instability, peroneus longus tendon autograft, thigh circumference

## Abstract

Background

Anterior cruciate ligament (ACL) reconstruction is among the most commonly performed knee procedures worldwide. Hamstring tendon autografts remain a popular graft choice but are associated with donor-site morbidity and postoperative thigh muscle weakness. The peroneus longus tendon has recently emerged as a promising alternative due to its favorable biomechanical strength and reportedly low ankle morbidity. However, detailed evidence comparing early functional recovery between these two graft options remains limited.

Objective

This study aims to compare the clinical and functional outcomes of ACL reconstruction using the peroneus longus tendon autograft versus the hamstring tendon autograft over a 6-month postoperative period.

Materials and methods

This prospective comparative study was conducted at a tertiary care center in India and included 50 patients with isolated ACL tears requiring primary single-bundle arthroscopic reconstruction. Participants were divided into two groups: Group A (peroneus longus autograft, n=25) and Group B (hamstring autograft, n=25) based on shared decision-making. Outcome measures included International Knee Documentation Committee (IKDC) scores, Lysholm scores, American Orthopaedic Foot and Ankle Society (AOFAS) scores, thigh circumference, ankle range of motion, and clinical knee stability tests. Assessments were performed preoperatively and at 1, 2, 3, and 6 months postoperatively. Statistical analyses utilized independent t-tests, paired t-tests, and chi-square tests or Fisher’s exact test with significance set at p<0.05.

Results

All 50 participants completed the 6-month follow-up. Both groups demonstrated excellent and comparable functional outcomes at 6 months, with significant improvements in IKDC and Lysholm scores, and knee stability outcomes showed grade 0 in ≥92% (≥46/50) of participants. The peroneus longus grafts showed a 14.7% greater mean diameter (8.94 mm vs. 7.79 mm; p<0.001). Thigh muscle preservation was significantly better in Group A, with 45.1% less thigh atrophy at 6 months (p<0.0001). AOFAS scores and ankle mobility were preserved equally in both groups at 6 months, and no graft failures occurred. One patient experienced mild knee stiffness, but no major complications were observed.

Conclusion

Peroneus longus autograft provides functional outcomes equivalent to hamstring autograft while offering a larger graft diameter and significantly better thigh muscle preservation, without compromising ankle function. These findings support its use as a safe and effective alternative, particularly advantageous for athletes or high-demand patients and in patients concerned about postoperative hamstring weakness.

## Introduction

The anterior cruciate ligament (ACL) is the most injured knee ligament, with 200,000-300,000 people sustaining ACL injuries each year in the United States [1]. ACL reconstruction has become the gold standard for symptomatic ACL-deficient knees, with an estimated 175,000 procedures performed per year worldwide [1,2]. Successful ACL reconstruction is critically important, and failure to restore adequate knee stability leads to knee instability, functional limitation of daily activities, and reduced sports participation [1,2]. Understanding the biomechanics and principles of ACL reconstruction guides optimal graft selection and determines the quality and durability of functional restoration [3].

Multiple autograft options are available for ACL reconstruction, including bone-patellar tendon-bone autograft, hamstring tendon (HT) autograft, quadriceps tendon autograft, and alternative graft sources such as the peroneus longus tendon (PLT) autograft [1,2]. Meta-analytic evidence shows that both patellar tendon and HT autografts are effective and popular choices for primary anterior cruciate ligament reconstruction (ACLR) [4]. A systematic review supported the viability and effectiveness of HT autografts for ACL reconstruction, establishing HT autograft as one of the favored autograft sources at various orthopedic centers [5]. HT autografts have been widely used and are the preferred graft source in many institutions worldwide due to the relative technical ease of harvest, predictable healing properties, and good reported long-term functional outcomes [1,5].

Although HT grafts are widely used and have proven effective, multiple important limitations and considerations should be discussed in detail when selecting the graft source best suited to an individual patient. HT harvest is associated with persistent and clinically significant donor site morbidity, including thigh muscle wasting, decreased knee flexor strength, and significant patient dissatisfaction [6]. The pathophysiological mechanism of this complication is the disruption of the musculotendinous continuity of the hamstring muscles during graft harvest, leading to denervation and disuse atrophy of these muscles, which play an important role in knee flexion and hip extension (providing dynamic knee stabilization during deceleration and pivoting and in athletes requiring explosive hamstring power) [6,7]. In addition, the diameter of the HT graft is often unpredictable and anatomically variable, requiring supplementation or augmentation with synthetic material to achieve the desired graft diameter for secure fixation in the tibial and femoral tunnels [4]. These limitations have led the orthopedic community to continuously search for alternative autograft sites that reduce donor-site morbidity while preserving the excellent functional outcomes demonstrated with hamstring grafting.

In recent years, the PLT has emerged as an alternative source of autograft for primary ACL reconstruction, with several theoretical and documented advantages over conventional hamstring grafts. The PLT has excellent biomechanical properties, with high load-to-failure strength and tensile properties similar to those of HTs and native ACL tissue [3,8]. Importantly, PLT harvest does not compromise or disrupt the hamstring musculature, thus theoretically eliminating significant donor-site morbidity of HT harvesting and full functional hamstring capability for postoperative rehabilitation and return to sports [1]. From an anatomical standpoint, multiple studies have shown that the peroneus brevis is the dominant and primary ankle evertor rather than the peroneus longus and is responsible for ankle stabilization and eversion strength [9]. The anatomical finding that peroneus longus harvest can be performed without clinically significant ankle morbidity or ankle instability is an important observation, a concern that has limited the use of peroneus longus as an autograft source in the past [9]. The use of the PLT as an autograft source has been proven in various orthopedic reconstructions beyond ACL reconstruction, such as ankle ligament, deltoid ligament, and spring ligament reconstructions in flatfoot deformity, without significant donor-site morbidity or functional compromise [7,10].

Meta-analytic evidence has confirmed peroneus longus and hamstring autografts to be functionally equivalent for ACL reconstruction [8]. Recently, a detailed prospective comparative study of 194 patients with functional equivalence between the two graft sources and better thigh muscle preservation in the peroneus longus group was reported [1]. A recent systematic review and meta-analysis of the comparative efficacy of peroneus longus versus hamstring autografts incorporated available evidence and confirmed the similar functional outcomes of the two graft sources [11]. However, there are limited studies on the detailed characterization of the temporal recovery pattern and progressive nature of thigh muscle atrophy at the critical early postoperative rehabilitation period [1,12]. Further comprehensive evaluation of ankle range-of-motion parameters and a detailed intraoperative comparison of graft characteristics have not been evaluated in other comparative studies. Furthermore, while most studies have followed patients for one year or more, early functional recovery, short-term follow-up, such as that occurring in the first 6 months, which is an important period for rehabilitation success and most important in returning to normal activities of daily living, has received comparatively little attention. Therefore, this prospective comparative study aimed to evaluate the clinical and functional outcomes after arthroscopic single-bundle ACL reconstruction using either the PLT or HT autograft and serial thigh circumference to describe the temporal pattern of donor-site morbidity and functional outcomes and donor-site complications at both the knee and ankle.

## Materials and methods

Study design and ethical approval

This prospective comparative observational study was conducted from 16 September 2024 to 15 September 2025 at the Department of Orthopaedics, Government Medical College and Rajindra Hospital, Patiala. Institutional Ethics Committee approval was obtained prior to study initiation (approval no.: Trg.8(109),2024/28172-189), and all procedures adhered to the Declaration of Helsinki and guidelines of the Indian Council of Medical Research (ICMR). Written informed consent was obtained from all participants in both English and Punjabi.

Patient selection

Adult patients aged 18-50 years presenting with isolated ACL rupture confirmed on magnetic resonance imaging (MRI) and requiring primary arthroscopic single-bundle reconstruction were consecutively screened.

Inclusion Criteria

Eligible patients were adults aged 18-50 years with an isolated ACL rupture on clinical examination, a primary ACL injury with no prior ligament surgery, no associated multi-ligament involvement, no previous surgery on either knee, or a complete ACL tear confirmed on MRI.

Exclusion Criteria

Patients were excluded if they had associated ligamentous injuries, chronic neuromuscular disorders, an active joint infection, inflammatory arthropathies, metabolic bone disease, neoplastic disease, or severe peripheral vascular disease.

Sample size

The final sample size consisted of 50 patients (25 per group). The absence of a pre-study power calculation is acknowledged as a limitation and may increase the risk of type II error for small between-group differences.

Surgical technique

All procedures were performed by a single experienced orthopedic surgeon in a standardized manner by arthroscopic single-bundle cruciate ligament reconstruction under spinal anesthesia. To confirm the diagnosis and grade of laxity without the influence of muscle guarding, a confirmatory Examination Under Anesthesia (EUA) (Lachman, Anterior Drawer, and Pivot Shift tests) was performed for every patient immediately following the administration of spinal anesthesia and prior to surgical incision. Data recorded from the EAU were used as a preoperative baseline for clinical stability measures. Diagnostic arthroscopy was performed using anteromedial and anterolateral portals to confirm a complete ACL tear and assess for concomitant intra-articular pathology (meniscal tears, articular cartilage damage, and other ligamentous injuries). Single-bundle reconstruction was performed using one tibial and one femoral tunnel corresponding to the site of the native ACL attachment. The femoral tunnel was fixed using a fixed-loop EndoButton, and the tibial tunnel was fixed using a biointerference screw.

For the peroneus longus, the graft was harvested through a 2 cm longitudinal incision over the posterior border of the lateral malleolus, and the peroneus longus was identified in the compartment beneath the peroneus brevis, isolated, and transected distally at the level of the lateral malleolus. The proximal tendon was released proximally (4-5 cm from the fibular head using a tendon stripper to avoid peroneal nerve injury), and the distal stump was sutured to the peroneus brevis. The tendon was separated from the muscular tissue and folded to form a triple-strand graft.

For the hamstring, the graft was harvested through a 2 cm oblique incision made 2 cm medial to the tibial tuberosity and 3 cm above the pes anserinus. The sartorial fascia was identified and incised longitudinally. Both semitendinosus and gracilis tendons were harvested using an open tendon stripper and folded to form a quadrupled-strand graft, which was secured using a number 2 polyester whip-stitch suture.

Outcome measures

Outcomes were assessed using the English versions of the International Knee Documentation Committee (IKDC) Subjective Knee Evaluation Form, the Lysholm Knee Scoring Scale, and the American Orthopaedic Foot and Ankle Society (AOFAS) Ankle-Hindfoot Score. For participants who were not fluent in English, a bilingual investigator verbally translated the questions into Punjabi at each follow-up to ensure consistency [13].

The IKDC subjective score was calculated according to standard scoring guidelines by summing item responses and transforming the raw value to a 0-100 scale, where higher scores indicate better knee function and fewer symptoms [14].

The Lysholm score was computed based on eight domains (limp, support, locking, instability, pain, swelling, stair climbing, and squatting) to yield a total score from 0 to 100. Interpretation was as follows: excellent (95-100), good (84-94), fair (65-83), and poor (<65) [15].

Donor-site morbidity at the ankle was evaluated using the AOFAS Ankle-Hindfoot score, which includes pain (40 points), function (50 points), and alignment (10 points), with total scores ranging from 0 to 100, where higher scores indicate better ankle-hindfoot function [16].

Knee range of motion (ROM) was assessed clinically at each follow-up. Restoration was considered successful if active flexion and extension were symmetric to the contralateral uninjured limb.

Postoperative protocol

A knee brace and limb elevation were used for immobilization. Intravenous antibiotics were administered for five days postoperatively. The wound was inspected on days 3, 5, and 7. Suture removal is done on days 12-15.

Rehabilitation protocol

Both groups underwent a standardized, accelerated rehabilitation program.

Phase 1 (0-2 Weeks)

The knee was immobilized in a brace in full extension. Patients performed ankle pumps and static quadriceps strengthening (quadriceps sets) immediately post-surgery. Partial weight-bearing with crutches was permitted. Gentle active-assisted ROM exercises (heel slides) were initiated to achieve 0-90° flexion.

Phase 2 (3-6 Weeks)

Progression to full weight-bearing was encouraged as tolerated. The brace was unlocked for ambulation. Closed kinetic chain exercises (mini-squats, 0-45°) were introduced. The goal was to achieve full active ROM comparable to the contralateral side.

Phase 3 (6 Weeks-3 Months)

Introduction of proprioceptive training (balance board) and open kinetic chain exercises. Strengthening of hamstrings and quadriceps was intensified.

Phase 4 (3-6 Months)

Agility drills, straight-line jogging, and plyometric exercises were initiated, with a gradual return to non-contact sports activities allowed after 6 months, pending functional clearance.

Statistical analysis

Data were analyzed using IBM SPSS Statistics for Windows, Version 26 (Released 2019; IBM Corp., Armonk, New York). The Shapiro-Wilk test was used to assess the normality of the data distribution. Continuous variables are presented as mean ± standard deviations. For between-group comparisons (Group A vs. Group B), independent t-tests were used. For within-group comparisons (preoperative vs. postoperative time points), paired t-tests were employed. Categorical variables were compared using the chi-square test or Fisher’s exact test. To address multiple comparisons across serial follow-ups (1, 2, 3, and 6 months), a Bonferroni correction was applied to the significance level. A p-value of less than 0.0125 (0.05/4) was considered statistically significant for these serial comparisons. For all other single-point comparisons, significance was set at p < 0.05.

## Results

A total of 50 patients underwent arthroscopic single-bundle ACLR. A total of 25 patients received PLT autografts (Group A), and 25 received HT autografts (Group B).

Baseline characteristics

The baseline characteristics of the patients are summarized in Table 1. There were no significant differences between groups in terms of age, gender distribution, or BMI. The PLT group had a significantly larger graft diameter than the HT group.

**Table 1:** Baseline demographic and graft characteristics of the study population PLT: Peroneus Longus Tendon graft group; HT: Hamstring Tendon graft group.

Variable	PLT (n=25)	HT (n=25)	Test Statistic	p-value
Age (years)	37.2±6.3	36.2±6.3	t=0.509	0.6132
Gender (M/F)	19/6	21/4	χ²=0.125	0.7237
BMI (kg/m²)	23.8±0.8	24.2±0.6	t=-1.842	0.0717
Graft Diameter (mm)	8.94±0.15	7.79±0.21	t=21.705	<0.0001

Knee function outcomes

IKDC Scores

Table 2 shows that preoperative IKDC scores were comparable between the groups (Group A: 52.0 ± 1.8 vs. Group B: 52.5 ± 2.0; t=-0.959, p=0.3421). At the 6-month follow-up, scores were 80.8±2.6 and 79.8±3.0 for Groups A and B, respectively (t=1.260, p=0.2138), with mean improvements of 28.8 and 27.3 points, respectively. No statistically significant between-group differences were observed at any time point.

**Table 2:** International Knee Documentation Committee (IKDC) scores (0-100 scale, higher indicates better knee function and fewer symptoms) from preoperative baseline through 6-month follow-up PLT: Peroneus Longus Tendon graft group; HT: Hamstring Tendon graft group.

Timepoint	PLT (n=25)	HT (n=25)	t-value	p-value
Preoperative	52.0±1.8	52.5±2.0	-0.959	0.3421
1 month	57.1±2.1	57.5±2.0	-0.678	0.5011
2 months	62.7±2.2	62.8±1.5	-0.220	0.8268
3 months	69.7±2.3	70.0±2.6	-0.401	0.6905
6 months	80.8±2.6	79.8±3.0	1.260	0.2138

Lysholm Knee Scoring Scale

Table 3 shows that preoperative Lysholm scores were comparable between groups (Group A: 53.4 ± 2.7 vs. Group B: 53.2 ± 2.3; t=0.280, p=0.7810). At the 6-month follow-up, scores were 96.0 ± 1.0 in Group A and 96.3 ± 1.0 in Group B (t=-0.831, p=0.4099). All time points showed no statistically significant between-group differences, indicating complete functional equivalence for knee stability outcomes between groups.

**Table 3:** Lysholm Knee Scoring Scale (0-100 scale, higher indicates better knee stability and fewer instability symptoms) from preoperative baseline through 6-month follow-up PLT: Peroneus Longus Tendon graft group; HT: Hamstring Tendon graft group.

Timepoint	PLT (n=25)	HT (n=25)	t-value	p-value
Preoperative	53.4±2.7	53.2±2.3	0.280	0.7810
1 month	61.0±2.5	60.8±2.2	0.351	0.7271
2 months	70.3±2.1	71.0±1.7	-1.276	0.2081
3 months	81.1±2.1	80.9±1.3	0.394	0.6952
6 months	96.0±1.0	96.3±1.0	-0.831	0.4099

Donor-site morbidity

AOFAS Scores

Table 4 shows that at the preoperative time point, there was no statistically significant difference between the PLT and HT groups, indicating comparable baseline values. At 1 and 2 months postoperatively, the HT group demonstrated significantly higher scores than the PLT group (p < 0.001), suggesting superior early outcomes in the HT group. At 3 and 6 months postoperatively, both groups showed similarly high scores, with no significant differences, indicating that outcomes between the two groups converged over time. Overall, the results suggest an early advantage for the HT group at 1 and 2 months, with comparable outcomes between the groups in the mid- to long-term follow-up.

**Table 4:** American Orthopaedic Foot and Ankle Society (AOFAS) scores (0-100 scale, higher indicates better ankle and foot function) assessing donor-site morbidity from graft harvest from preoperative baseline through 6-month follow-up PLT: Peroneus Longus Tendon graft group; HT: Hamstring Tendon graft group.

Timepoint	PLT Group (n=25)	HT Group (n=25)	t-value	p-value
Preoperative	96.1±0.8	96.2±0.9	-0.236	0.8142
1 Month	88.4±3.5	95.8±1.2	-9.4	<0.001
2 Months	93.2±2.1	96.5±1.0	-7.09	<0.001
3 Months	98.1±0.8	98.0±0.9	0.41	0.68
6 Months	99.5±0.5	99.7±0.3	-1.682	0.099

Thigh circumference difference

The difference in thigh circumference between the injured and uninjured limbs was similar at baseline (Group A: 1.801±0.051 cm vs. Group B: 1.816±0.063 cm; t=-0.881, p=0.3828). At the 6-month follow-up, the thigh circumference difference was 0.751 ± 0.065 cm in Group A and 1.367 ± 0.087 cm in Group B (t=-27.852, p<0.0001), representing significantly better preservation of thigh muscle bulk in the peroneus longus group compared with the hamstring group (Table 5).

**Table 5:** Thigh circumference difference (injured vs. uninjured limb) in centimeters from preoperative baseline through 6-month follow-up. Lower values indicate better muscle preservation with less atrophy PLT: Peroneus Longus Tendon graft group; HT: Hamstring Tendon graft group.

Timepoint	PLT (n=25)	HT (n=25)	t-value	p-value
Preoperative	1.801±0.051	1.816±0.063	-0.881	0.3828
1 month	1.622±0.063	1.736±0.058	-6.586	<0.0001
2 months	1.426±0.057	1.660±0.070	-12.624	<0.0001
3 months	1.112±0.064	1.517±0.058	-22.937	<0.0001
6 months	0.751±0.065	1.367±0.087	-27.852	<0.0001

ROM

Ankle and Hindfoot ROM at the 6-Month Follow-Up

The ankle and hindfoot ROM in both groups at 6-month follow-up was not statistically significantly different across all planes. Dorsiflexion was 20.6 ± 2.8° (Group A) versus 20.5 ± 3.0° (Group B) (t=0.175, p=0.8617). Plantarflexion was 36.9 ± 1.8° (Group A) and 37.6 ± 1.6° (Group B) (t=-1.581, p=0.1205). Inversion was 29.4 ± 2.0° (Group A) and 30.1 ± 1.7° (Group B) (t=-1.406, p=0.1662). Eversion was 24.0±2.5° (Group A) and 24.2±2.0° (Group B) (t=-0.277, p=0.7833) (Table 6).

**Table 6:** Ankle and hindfoot range of motion in four planes at 6-month follow-up Data are presented as mean ± standard deviation in degrees, with normal reference ranges provided. PLT: Peroneus Longus Tendon graft group; HT: Hamstring Tendon graft group.

ROM Plane	PLT (n=25)	HT (n=25)	Normal Range	t-value	p-value
Dorsiflexion	20.6±2.8°	20.5±3.0°	15-25°	0.175	0.8617
Plantarflexion	36.9±1.8°	37.6±1.6°	35-50°	-1.581	0.1205
Inversion	29.4±2.0°	30.1±1.7°	25-35°	-1.406	0.1662
Eversion	24.0±2.5°	24.2±2.0°	15-25°	-0.277	0.7833

Clinical knee stability tests

Anterior Drawer Test

Table 7 presents the anterior drawer test results. Preoperatively, the majority of patients in both Group A and Group B demonstrated gross instability (Grades 2-3). At the 6-month follow-up, there was marked improvement in both groups, with 96% of patients showing normal findings (Grade 0) and only 4% showing mild translation (Grade 1). No patients had moderate or gross instability at 6 months in either group, indicating excellent postoperative stabilization with comparable outcomes between the groups.

**Table 7:** Anterior drawer test results at preoperative and 6-month follow-up Data are presented as numbers (percentages). Between-group comparison (Group A vs. Group B): Preoperative p = 1.000; 6 months p = 1.000. Statistical test: Chi-square test/Fisher's exact test. Group A: Peroneus longus tendon (PLT) graft group; Group B: Hamstring tendon (HT) graft group.

Grade	Interpretation	Group A Preoperative	Group A 6 Months	Group B Preoperative	Group B 6 Months
0	Negative/normal	0 (0%)	24 (96%)	0 (0%)	24 (96%)
1	Mild translation	0 (0%)	1 (4%)	0 (0%)	1 (4%)
2	Moderate translation	6 (24%)	0 (0%)	5 (20%)	0 (0%)
3	Gross instability	19 (76%)	0 (0%)	20 (80%)	0 (0%)

Lachmann Test

Table 8 shows that both groups showed marked improvements in anterior knee stability after surgery. Preoperatively, most patients in both groups had severe laxity, but by 6 months, all cases of moderate and severe laxity had resolved. Group A achieved normal stability in 96% of patients, and Group B in 92%, with only mild residual laxity in a few patients. Overall, both grafts provided excellent restoration of anterior stability.

**Table 8:** Lachmann test results at preoperative and 6-month follow-up Data are presented as numbers (percentages). Between-group comparison (Group A vs. Group B): Preoperative p = 1.000; 6 months p = 1.000. Statistical test: Chi-square test/Fisher's exact test. Group A: Peroneus longus tendon (PLT) graft group; Group B: Hamstring tendon (HT) graft group.

Grade	Interpretation	Group A Preoperative	Group A 6 Months	Group B Preoperative	Group B 6 Months
0	Negative/normal	0 (0%)	24 (96%)	0 (0%)	23 (92%)
1	Mild laxity	0 (0%)	1 (4%)	0 (0%)	2 (8%)
2	Moderate laxity	3 (12%)	0 (0%)	4 (16%)	0 (0%)
3	Severe laxity	22 (88%)	0 (0%)	21 (84%)	0 (0%)

Pivot Shift Test

Table 9 shows that both groups demonstrated complete improvement on the pivot shift test, indicating restoration of rotational knee stability at the 6-month follow-up. Preoperatively, all patients in Group A and Group B tested positive for rotational instability (100%). By 6 months, every patient in both groups demonstrated a negative test (100%), indicating full restoration of anteriolateral rotational knee stability with either graft choice.

**Table 9:** Pivot shift test results at preoperative and 6-month follow-up Data are presented as numbers (percentages). Between-group comparison (Group A vs. Group B): Preoperative p = 1.000; 6 months p = 1.000. Statistical test: Chi-square test/Fisher's exact test. Group A: Peroneus longus tendon (PLT) graft group; Group B: Hamstring tendon (HT) graft group.

Result	Group A Preoperative	Group A 6 Months	Group B Preoperative	Group B 6 Months
Positive	25 (100%)	0 (0%)	25 (100%)	0 (0%)
Negative	0 (0%)	25 (100%)	0 (0%)	25 (100%)

Complications

Table 10 shows that complication rates were low in both groups. Group A had one complication (mild knee stiffness, 4%), whereas Group B had no complications (0%). By the 6-month follow-up, 98% of patients (49/50) had regained a full active ROM comparable to the uninjured limb. The single patient in Group A had mild stiffness with a flexion deficit of <10° compared with the contralateral knee and was managed conservatively. No graft failures were observed in either group during the 6-month follow-up period.

**Table 10:** Postoperative complications through 6-month follow-up Group A: Peroneus longus tendon (PLT) graft group; Group B: Hamstring tendon (HT) graft group.

Complication	Group A (n=25)	Group B (n=25)
Mild knee stiffness	1 (4%)	0 (0%)
Graft failure	0 (0%)	0 (0%)
Total complications	1 (4%)	0 (0%)

## Discussion

This prospective comparative study of 50 patients showed that the PLT autograft provides functional outcomes equivalent to those of the HT autograft at 6-month follow-up.

IKDC scores were excellent in both groups, with Group A (PLT) achieving 80.8±2.6 and Group B (HT) achieving 79.8±3.0, with no statistically significant difference (t=1.260, p=0.214). Similarly, Lysholm Knee Scores were comparable between groups, with Group A achieving 96.0±1.0 and Group B achieving 96.3±1.0, without a statistically significant difference (t=-0.831, p=0.410). Both groups demonstrated substantial improvements from baseline. These results align with the recent large 194-patient study by Agarwal et al., which also demonstrated functional equivalence between similar graft sources, as indicated by comparable scores at the 1-year follow-up period [10]. Meta-analytic evidence by He et al. confirms functional equivalence at the population level, supporting the clinical viability of the peroneus longus graft [4]. Clinical knee stability testing revealed excellent restoration, with 96% negative anterior drawer tests, 92%-96% negative Lachman testing, and 100% negative pivot shift testing (all p > 0.05), indicating equivalent anterolateral stability restoration with both graft resources [3].

Thigh muscle preservation was better with peroneus longus grafting during the 6-month follow-up period. The thigh circumference difference (measured 15 cm proximal to the patella) progressively decreased in both groups, but the preservation of the peroneus longus in Group A was significantly better at all times. The thigh circumference difference (measured 15 cm proximal to the patella) was equivalent at baseline (Group A: 1.801±0.051 vs. Group B: 1.816±0.063, p=0.383), but progressively diverged with significantly better preservation in the PLT group at all postoperative timepoints: 1 month (1.622±0.063 vs. 1.736±0.058 cm, p<0.0001); 2 months (1.426±0.057 vs. 1.660±0.070, p<0.0001); 3 months (1.112±0.064 vs. 1.517±0.058 cm, p<0.0001); and 6 months (0.751±0.065 vs. 1.367±0.087 cm, p<0.0001). This represents a significantly lower reduction in thigh circumference in the peroneus longus group, a highly statistically significant and clinically meaningful advantage in cosmesis and functional recovery for the patient.

Similarly, Agarwal et al. reported superior thigh preservation at 1-year follow-up (0.216 vs. 0.88 cm), suggesting advantages beyond 6 months [10]. This temporal characterization across all five time points provides evidence of superior preservation that begins in the earliest postoperative period and progressively increases, based on basic anatomical differences. The semitendinosus and gracilis tendons are denervated, and subsequent atrophy occurs due to the disruption of the musculotendinous junction of the muscle during HT harvest. Tashiro et al. demonstrated that this leads to chronic deficits in knee flexor strength [17]. Thomas et al. further showed that muscle atrophy is the primary mechanism, involving denervation of muscle fibers distal to the harvest site and disuse atrophy [7]. In contrast, peroneus longus harvest does not interfere with the hamstring musculature, preserving neurological innervation and musculotendinous continuity, allowing the hamstring muscles to participate in the early postoperative rehabilitation period and reducing atrophy.

Clinically, this 45% reduction in thigh atrophy is significant for athletes and active patients who rely on hamstring function for activities that require acceleration and deceleration, as well as rotational activities. The preservation of hamstring strength is critical for the longevity of the ACL graft. Biomechanical studies have established that the hamstrings act as dynamic ACL agonists, providing a posterior tibial force that protects the graft during high-risk maneuvers such as rapid deceleration and landing [18,19]. Consequently, HT harvesting may compromise this dynamic restraint, potentially increasing the risk of graft failure. Furthermore, persistent thigh muscle atrophy is not merely a cosmetic concern but a functional one. Significant deficits in hamstring volume and strength have been correlated with altered landing biomechanics and a higher risk of re-injury upon return to sport [20,21]. By utilizing the PLT, our study demonstrates significantly better preservation of thigh muscle bulk, which may theoretically enhance the dynamic stability of the knee. In the non-athletic population, preserving hamstring function is important for normal gait mechanics, climbing stairs, and daily functional capacity [22,23]. Peroneus longus grafts had a significantly larger mean diameter (8.94 ± 0.15 mm vs. 7.79 ± 0.22 mm, p < 0.0001), which is equivalent to a 14.7% advantage that eliminates the need for supplementation with synthetic material, which is necessary with smaller hamstring grafts [24,25].

It is important to remember that the peroneus brevis muscle, not the peroneus longus muscle, is the main ankle evertor [8], which can impact the safety of the peroneus longus harvest. Consequently, the peroneus longus harvest did not result in ankle instability or major ankle functional limitation. This is confirmed by ankle ROM: dorsiflexion (20.6 ± 2.8 vs. 20.5 ± 3.0 deg, p=0.862), plantarflexion (36.9 ± 1.8 vs. 37.6 ± 1.6 deg, p=0.121), inversion (29.4 ± 2.0 vs. 30.1 ± 1.7 deg, p=0.166), and eversion (24.0 ± 2.5 vs. 24.2 ± 2.0 degrees, p=0.783) with no significant statistical difference [9,17,24].

Recent systematic reviews have provided sufficient support. Park et al. (2024) confirmed functional equivalence between grafts with peroneus longus advantages in donor-site morbidity [2]. Functional equivalence was confirmed at the population level in a meta-analysis by He et al. (2020) [4]. Rhatomy et al. (2019) [24] specifically recommended peroneus longus as a superior graft, citing superior thigh muscle preservation. Gok et al. (2024) compared 106 patients with a 1-year follow-up, documenting functional equivalence with substantially better thigh circumference preservation, which is very similar to the results of this study, despite the larger numbers and longer follow-up [26]. Overall complication rates were low and equivalent, with no graft failures or neurovascular complications reported in either group, further establishing the safety of both techniques.

The strengths of this study include its prospective design, 100% follow-up, well-matched groups, and consistent surgical technique, as all procedures were performed by a single senior surgeon, improving internal validity. Limitations include the single-center, single-surgeon setting, which may reduce generalizability, the short 6-month follow-up, and assessment based mainly on circumference measurements without advanced imaging. Data regarding specific sports activities, pre-injury activity levels, and chronicity of injury were not recorded for this study. A validated written Punjabi version of the IKDC and Lysholm scores was unavailable, so standardized verbal translation was used, which may introduce minor variability. Because return to sport typically occurs at 9-12 months, longer and multicenter studies with return-to-sport analysis are needed to determine long-term performance outcomes.

Clinically, the peroneus longus autograft can be considered a safe and potentially better alternative for primary ACL reconstruction in competitive high-demand patients, patients who are concerned about postoperative thigh weakness, and those requiring an optimal graft diameter. A peroneus longus harvest should be performed as part of routine practice, and patient counseling should emphasize donor-site morbidity, especially the benefits of preserving the thigh muscle.

## Conclusions

This prospective comparative study showed that the PLT autograft provides functional outcomes equivalent to those of the HT autograft at 6 months of follow-up for ACL reconstruction. Importantly, peroneus longus grafting led to significantly less circumferential loss, as evidenced by a progressive advantage in thigh circumference measurements from as early as 4 weeks postoperatively to 6 months of follow-up. This superior donor-site outcome has clinical importance for patient satisfaction and for high-demand patients who require preserved hamstring function for participation in sports. Combined with the biomechanical advantage of a considerably larger graft diameter, good preservation of ankle joint function despite the harvested ankle donor site, and comparable safety profiles between graft sources, these findings suggest the peroneus longus autograft is a viable alternative for primary ACL reconstruction, especially in patients who are concerned about postoperative thigh weakness or in competitive high-demand patients. The PLT is a safe option that offers comparable knee function to the HT with the added benefit of reduced thigh muscle atrophy to the HT autograft for arthroscopic single-bundle ACL reconstruction.
